# SVNeoPP: A Workflow for Structural-Variant-Derived Neoantigen Prediction and Prioritization Using Multi-Omics Data

**DOI:** 10.3390/biology15060492

**Published:** 2026-03-19

**Authors:** Wanyang An, Xiaoxiu Tan, Zhenhao Liu, Li Zou, Manman Lu, Lu Xie

**Affiliations:** 1 School of Health Science and Engineering, University of Shanghai for Science and Technology, Shanghai 200093, China; 232322231@st.usst.edu.cn (W.A.); 242322232@st.usst.edu.cn (L.Z.); 2 Shanghai-MOST Key Laboratory of Health and Disease Genomics, Shanghai Institute for Biomedical and Pharmaceutical Technologies, Shanghai 200237, China; tanxiaoxiu@sibpt.cn (X.T.); liuzhenhao@sibpt.cn (Z.L.); lumanman@sibpt.cn (M.L.); 3 College of Food Science and Technology, Shanghai Ocean University, Shanghai 201306, China; 4 Shanghai Institute for Biomedical and Pharmaceutical Technologies, School of Public Health, Fudan University, Shanghai 200237, China

**Keywords:** neoantigens, structural variants, prediction workflow, multi-omics, Snakemake

## Abstract

Background: Tumor neoantigens are key targets for personalized vaccines and T-cell therapies, yet most pipelines focus on neoantigens derived from SNV/small indel and often yield a limited number of high-quality candidates. SVs are prevalent in tumors and can generate novel chimeric sequences and neopeptides, making them a promising additional source of neoantigens. However, SV-derived neoantigen prediction remains challenging due to breakpoint uncertainty, isoform-dependent coding inference, and limited integration of multi-dimensional evidence and reproducibility. Methods: We developed SVNeoPP (Structural Variant Neoantigen Prediction and Prioritization), an end-to-end workflow for SV-derived neoantigen analysis. SVNeoPP takes WGS and RNA-seq as inputs, performs SV calling and annotation, and reconstructs altered transcripts and coding sequences in a traceable, isoform-aware manner to generate candidate peptides. Candidates are prescreened by integrating antigen-processing features with HLA binding prediction, and then hierarchically filtered and prioritized based on transcript expression, LC–MS/MS proteomics evidence, immunogenicity predictions, and sequence similarity to experimentally validated neoantigen databases. SVNeoPP is implemented in Snakemake to enable modular extension, checkpoint-based restarts, and end-to-end reproducibility. Results: Using a hepatocellular carcinoma (HCC) multi-omics dataset as a proof of concept, we demonstrated the performance of SVNeoPP and obtained a high-priority shortlist of candidate peptides. Compared with other methods, SVNeoPP substantially expanded the candidate search space for SV-derived neoantigens and showed more favorable distributions of antigen-processing and HLA binding features. Conclusions: SVNeoPP provides a reusable, traceable, and interpretable multi-dimensional evidence-driven framework for SV-derived neoantigens. As a complementary module to SNV/small-indel pipelines, it broadens the neoantigen candidate repertoire and generates ranked candidates with interpretable evidence to facilitate downstream prioritization and decision-making.

## Simple Summary

Structural variants (SVs) are widespread in tumors and can generate protein fragments with larger tumor–normal differences through genomic rearrangements or gene fusion events, making them an important source of neoantigens. In this study, we developed SVNeoPP (Structural Variant Neoantigen Prediction and Prioritization), a reproducible analytical workflow that converts SV-breakpoint information into peptide sequences, predicts candidate neoantigens, and prioritizes them through stepwise filtering supported by multi-dimensional evidence, including transcriptomic and proteomic data. In a proof-of-concept application to hepatocellular carcinoma (HCC) multi-omics datasets, SVNeoPP produced a high-confidence, high-priority shortlist of candidate neoantigens. SVNeoPP may be complemented as a useful bioinformatics tool for human genome structure and function analysis.

## 1. Introduction

Recent advances in cancer immunotherapy have highlighted the therapeutic potential of neoantigen-based personalized vaccines and T-cell therapies. Neoantigens originate from mutant proteins generated by tumor-specific somatic alterations. Peptides produced through proteasomal degradation can bind major histocompatibility complex (MHC) molecules (human leukocyte antigen, HLA, in humans) and be presented on the cell surface as peptide–HLA complexes, where they are recognized by T cells to trigger antitumor immune responses [1]. Since tumor-specific neoantigens are less subject to central tolerance and are generally considered less likely to induce autoimmunity, they are viewed as safe and highly promising therapeutic targets [2]. Recent early-phase clinical studies have indicated that personalized neoantigen vaccines can elicit tumor-specific immune responses and may reduce the risk of recurrence [3,4,5]. Therefore, accurately identifying and prioritizing neoantigens with immunogenicity is considered essential for enhancing the efficacy of neoantigen-based immunotherapies.

Computational discovery and prioritization of neoantigens typically involve variant calling, peptide generation, peptide–HLA binding prediction, and immunogenicity assessment [6]. Existing studies have primarily focused on neoantigens derived from SNVs/small indels [7], and various prediction methods have been developed. For example, our previous work utilized ProGeo-neo and PGNneo to integrate genomics, transcriptomics, and proteomics to identify candidate neoantigens from both coding and non-coding regions [8,9]. However, focusing solely on neoantigens derived from SNVs/small indels has been shown to limit the number of high-quality candidates and to constrain tumor specificity and immunogenicity, thereby restricting their translational potential [10]. Structural variants (SVs) represent another important type of alterations in cancer and are prevalent in approximately 94.9% of tumor types [11]. SVs involve genomic rearrangements larger than 50 bp, spanning events from single genes to whole chromosomes, including deletions, insertions, inversions, duplications, translocations and other breakends [12]. These events can cause frameshifts, generate novel C-terminal sequences, or create fusion transcripts/fusion proteins, thus introducing de novo sequences that differ significantly from normal proteins. Due to their novelty and ubiquity, SV-derived sequences are considered an important complementary source of neoantigens.

Despite the expanded potential tumor antigen scope, the computational identification of SV-derived neoantigens involves a complex inference chain. First, SV breakpoint calling and event annotation are subject to uncertainty: in a multi-transcript context, the same breakpoint may result in different coding outcomes, highlighting the need for standardized and traceable strategies for transcript and coding-sequence reconstruction [13]. Second, prioritizing candidate neoantigens cannot rely solely on HLA binding predictions: it also requires integrating expression levels, antigen processing features, immunogenicity, and other factors. In the absence of multi-dimensional evidence, it is often difficult to effectively identify and filter high-confidence candidate neoantigens [14]. Therefore, an SV-derived neoantigen analysis framework should integrate variant interpretation and sequence derivation with downstream immunologic evaluation in an evidence-driven workflow, thereby reducing false positives and improving prioritization interpretability [15]. Although existing tools (e.g., NeoSV) have demonstrated the feasibility of mining neoantigens from SVs, limitations remain in end-to-end input support, workflow reproducibility, and multi-evidence integration [7,10]. Accordingly, there remains a need for an end-to-end framework that integrates traceable transcript and coding-sequence reconstruction with multi-dimensional evidence-driven prediction and prioritization of candidate SV-derived neoantigens.

To this end, we developed SVNeoPP, an integrated framework for SV-derived neoantigen discovery. SVNeoPP takes raw WGS and RNA-seq data as input to perform SV calling and annotation, and provides a standardized, traceable strategy for transcript and coding-sequence reconstruction in a multi-transcript context. This enables the reconstruction of SV-associated coding sequences and the derivation of corresponding candidate peptides. SVNeoPP then filters candidates using antigen processing features and predicts peptide–HLA binding. To improve reliability and interpretability, SVNeoPP further supports multi-dimensional prioritization by integrating RNA expression evidence, LC–MS/MS proteomics detection, immunogenicity prediction, and similarity to experimentally validated neoantigen databases. SVNeoPP is implemented in Snakemake, enabling modular extension and checkpoint restarts, and facilitating stable, reproducible end-to-end analyses for multi-omics inputs [16]. We applied SVNeoPP to multi-omics data from hepatocellular carcinoma (HCC) samples and performed comparative evaluation against NeoSV to demonstrate its effectiveness. Overall, this study provides a reproducible framework for computational identification of SV-derived neoantigens and multi-dimensional evidence integration, yielding a higher-confidence prioritized shortlist of candidate neoantigens.

## 2. Materials and Methods

SVNeoPP integrates SV calling and candidate peptide generation from whole-genome sequencing (WGS), HLA class I typing and expression quantification from RNA sequencing (RNA-seq), and proteomic evidence from LC–MS/MS to predict and prioritize SV-derived candidate neoantigens. The workflow is implemented using Snakemake (v9.4.1), enabling modular and reproducible computational analyses. The software tools and versions used in SVNeoPP are summarized in Table 1.

### 2.1. Data Collection

To demonstrate the applicability of SVNeoPP in a real-world cohort, we analyzed multi-omics data from four HCC patients, including tumor and matched normal samples with paired WGS, RNA-seq and LC–MS/MS proteomics data. This dataset was derived from a previously published study in which our group participated [17]. RNA-seq data are available from the GEO database (accession: GSE124535), and LC–MS/MS proteomics data are available from the iProX database (accession: IPX0000937000). The raw WGS data are not publicly available due to data-sharing restrictions but are available upon reasonable request and approval. Detailed information on data access and code availability is provided in the Data Availability Statement. In addition, we used multiple reference resources (e.g., reference genome and human proteome), as detailed in Table 2.

### 2.2. SV-Driven Candidate Peptide Construction

**Figure 1:**
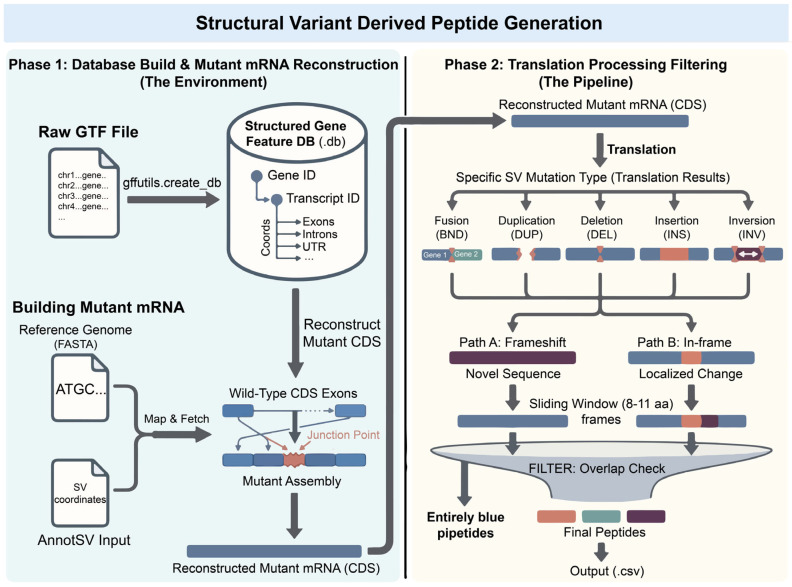
Schematic of SV-derived candidate peptide generation. The workflow has two steps: (1) A gene-feature index is built from GTF annotations using gffutils; wild-type CDS exons are reassembled according to SV breakpoint coordinates (reference FASTA + breakpoints) to reconstruct a mutant CDS/mRNA coding sequence containing a junction (red). (2) Mutant CDSs are translated into protein sequences covering multiple SV types (BND, DEL, DUP, INS, INV) and classified as frameshift events (introducing downstream novel sequence; purple) or in-frame events (local junction changes; red/cyan). Candidate peptides are extracted from the variant region with an 8–11-aa sliding window, followed by stringent self-peptide filtering to remove peptides identical to the reference sequence (blue). Only junction-spanning peptides or peptides within the novel sequence region are retained and exported as a CSV file.

This section describes the core module of SVNeoPP for generating SV-derived peptides. An overview of the algorithm is shown in Figure 1 and comprises three main steps: WGS preprocessing, somatic SV calling and annotation, and transcript-structure-guided generation of SV-derived peptides.

#### 2.2.1. WGS Preprocessing

First, WGS FASTQ reads from tumor and matched normal samples underwent quality control and preprocessing using fastp (v1.0.1; default parameters), including adapter trimming, low-quality base trimming, and filtering, to generate cleaned reads for alignment [18]. The cleaned reads were then aligned to the human reference genome hg38 using BWA-MEM (v0.7.19), generating alignment files [19], and the resulting alignments were processed with SAMtools (v1.22.1) for format conversion, sorting, and indexing [20].

To mitigate the impact of PCR duplicates on downstream variant detection, duplicate reads were marked using MarkDuplicates in GATK (v4.6.2.0). Base quality score recalibration (BQSR) was performed using BaseRecalibrator and ApplyBQSR in GATK (v4.6.2.0), with known variant sites from dbSNP (build 146) and the Mills and 1000 Genomes gold-standard indel as calibration resources [21]. The resulting recalibrated BAM files from tumor and matched normal samples served as inputs for somatic SV calling.

#### 2.2.2. SV Calling and Annotation

Somatic SV calling was performed on the recalibrated tumor–normal BAM files using SvABA (v1.2.0) [22] in multi-threaded mode with recommended parameters. dbSNP indel information was provided via the “-D” option to facilitate model construction and variant filtering. The resulting SV calls were output in VCF format and annotated using AnnotSV (v3.4.6) [23] in split mode, generating gene-specific records (one row per affected gene) to facilitate per-gene quantification and downstream analyses. Only SV events with FILTER = PASS in the VCF were retained.

To focus on SVs likely to affect coding sequences, only events overlapping transcript coding sequences (CDS) were retained. Specifically, an SV was considered overlapping the CDS if: (1) both breakpoints were within the CDS; (2) one breakpoint was within the CDS and the other outside; or (3) both breakpoints were outside the CDS, but the SV interval spanned the CDS, thereby deleting or altering part or all of the exon(s) (for interval-based SVs). The resulting gene-split functional annotation outputs were used to link SV breakpoints to transcript structures and served as inputs for mutant CDS reconstruction and SV-derived peptide generation.

#### 2.2.3. SV-Derived Peptide Generation

Based on SV calls and their annotations, SV events were mapped onto transcript structures, and mutant CDSs were reconstructed to generate SV-derived peptides. Exon/CDS coordinates and reading-frame information were extracted from GENCODE (release 47; gencode.v47.annotation.gtf) to enable transcript-level localization of SV breakpoints. Major SV categories (e.g., fusion-forming rearrangements, deletions, duplications, insertions, and inversions) were classified as frameshift or in-frame depending on coding-frame disruption. Operationally, for single-event SVs, AnnotSV frameshift annotation was prioritized when available; otherwise, frameshift status was determined by whether the net coding length was not a multiple of three. For fusion events, frameshift status was evaluated based on whether the nucleotide length of the 5′ (head) segment was divisible by three.

Reconstructed mutant CDSs were translated into amino acid sequences, and 8–11-amino-acid peptides were generated using a sliding window. To improve neoantigen specificity, only peptides spanning variant junctions or lying within SV-altered regions were retained, and peptides identical to the wild-type sequence were removed. The final candidate peptide list, annotated with SV and transcript information, served as input for HLA binding prediction, expression assessment, MS database searching, and integrated prioritization.

### 2.3. HLA Typing

Because peptide–HLA binding affinity is allele-dependent, accurate HLA typing is essential. HLA class I genotypes (HLA-A, HLA-B, and HLA-C) were inferred for each sample from trimmed tumor RNA-seq reads using OptiType (v1.3.5; default parameters) [24]. OptiType has been reported to achieve an HLA typing accuracy of approximately 97% [25].

### 2.4. Prescreening for Processing Potential and Peptide–HLA Binding Prediction

To account for the impact of proteasomal processing and the generation of presentable termini on peptide–HLA binding, SV-derived 8–11 aa peptides were prescreened for processing potential prior to binding prediction.

#### 2.4.1. NetChop Scoring and Internal Cleavage Risk Assessment

NetChop (v3.1; C-terminal model) was used to predict peptide cleavage probabilities, producing cleavage scores at each amino-acid position for every peptide [26]. For each peptide, the predicted scores at the N- and C-terminal positions were recorded as *N_score* and *C_score*, respectively. Internal positions (excluding both termini) with scores ≥ 0.5 were considered potential internal cleavage sites. Two summary metrics were then derived: the maximum internal cleavage score (*max_internal_score*) and the number of internal cleavage sites (*internal_cleavage_count*). Internal cleavage risk (*Internal_Risk*) was defined as follows:

(1) Internal_Risk= α × max_internal_score+ β × log(1 + internal_cleavage_count).

Here, α and β are predefined weights (α=1.0 and β=0.3 in this study), selected based on both biological rationale and grid-search calibration using a large set of SV-derived candidate peptides. From a biological perspective, *max_internal_score* was assigned a higher weight because, for short 8–11-mer peptides, even a single high-probability internal cleavage event may substantially disrupt peptide integrity [27]. In contrast, *internal_cleavage_count* was treated as an auxiliary feature and assigned a lower weight to avoid over-penalizing partially overlapping internal cleavage signals. Grid-search calibration further supported this parameterization and suggested that α=1.0 and β=0.3 provided a balanced setting for internal cleavage penalization (Supplementary Tables S1 and S2). In addition, our method allows users to conveniently adjust the α and β parameters through the configuration file (config.yaml), enabling the workflow to be adapted for specific protein families or customized analytical requirements. To facilitate distribution comparison and visualization across peptides/tools, we applied min–max normalization to *Internal_Risk* to obtain *Internal_Risk_norm*:

(2) $$Internal\_Risk\_norm\;=\;\frac{Internal\_Risk\;-\;{Internal\_Risk}_{min}}{{Internal\_Risk}_{max}\;-\;{Internal\_Risk}_{min}}.$$

By jointly considering terminal cleavage probabilities and internal cleavage risk, we defined an overall score (*NetChop_Score*) as follows:

(3) NetChop_Score = (N_score + C_score)/(1 + Internal_Risk).

A *NetChop_Score* ≥ 0.5 was used as a prescreening threshold to preferentially retain peptides with higher terminal cleavage potential and lower internal cleavage risk. This cutoff is consistent with the default NetChop 3.1 baseline for a positive cleavage prediction. In addition, sensitivity analysis further showed that, within our integrated scoring framework, this threshold provides a relatively balanced filtering criterion between terminal cleavage support and internal cleavage risk (Supplementary Table S3).

#### 2.4.2. Peptide–HLA Binding Prediction

After prescreening for antigen processing potential, peptide–HLA binding was evaluated using NetMHCpan (v4.1b) [28] and MHCflurry (v2.1.5) [29]. For NetMHCpan, peptide–allele pairs with BindLevel of “SB” or “WB” were considered to satisfy the criterion (net_pass). For MHCflurry, pairs with an affinity < 500 nM and percentile rank < 2.0 were considered to satisfy the criterion (flurry_pass). A peptide was included in the candidate neoantigen set if it satisfied net_pass or flurry_pass for at least one HLA allele of the corresponding sample.

### 2.5. Prioritization of Candidate Neoantigens Using Multi-Dimensional Features

To prioritize SV-derived neoantigen peptides with higher immunogenicity, evidence-informed filtering and prioritization were performed by integrating multi-omics information. The integrated features included RNA-seq expression levels of the source genes (TPM), LC–MS/MS database-searched evidence, predicted immunogenicity, and sequence similarity to experimentally validated neoantigens.

#### 2.5.1. RNA-Seq Quantification and Expression-Based Filtering

RNA-seq data were used to evaluate the expression of the source genes for candidate neoantigens. A reference transcriptome index was built with kallisto (v0.51.1) using GENCODE transcript annotations (release 47), followed by pseudoalignment and transcript abundance quantification [30]. Transcript abundances were aggregated to the gene level with tximport. The aggregated gene-level TPM > 0 was used to indicate detectable expression for filtering candidate neoantigens [31], while the aggregated gene-level estimated counts were exported simultaneously to serve as the raw input for downstream differential expression analysis.

#### 2.5.2. Personalized Search Database Construction and LC–MS/MS Proteomic Evidence

To provide proteomics-level evidence for SV-derived candidates, we constructed a personalized search database and performed database searching on the paired LC–MS/MS data. The personalized database comprised three components: (1) the candidate peptide set retained after NetChop prescreening, peptide–HLA binding prediction, and gene-level expression filtering; (2) a human reference protein sequence database; and (3) a common contaminant sequence database [32].

LC–MS/MS data were searched using the FragPipe (v23.1) workflow with MSFragger (v4.3) [33]. Enzymatic digestion was set to strict trypsin (fully tryptic cleavage at K/R sites), allowing up to two missed cleavages, with a minimum peptide length of 7 aa. Carbamidomethylation of cysteine was set as a fixed modification, and variable modifications included methionine oxidation and protein N-terminal acetylation. Mass tolerances were set to ±20 ppm for precursors and 20 ppm for fragments. Identifications were controlled using a target–decoy strategy, with false discovery rate (FDR) controlled at 1% at the PSM, peptide, and protein levels, respectively [34].

Peptide-level evidence was extracted from the combined_peptide.tsv output. Candidate peptides were selected if the “Protein” field contained the “neo_” prefix, labeling SV-derived entries in the personalized FASTA. To ensure tumor specificity, peptides mapping to the normal reference proteome were removed. Because neoantigen identification is fundamentally peptide-centric, peptides mapping to multiple “neo_” entries were retained. In our personalized SV-derived FASTA, such multi-mapping typically arises because the same mutant peptide sequence can be represented across multiple transcript isoforms or closely related SV-derived entries from the same event. The identification confidence of all such retained peptides was strictly safeguarded by the 1% peptide-level FDR threshold applied during the FragPipe workflow. All retained peptides required at least one supporting spectrum (Spectral Count > 0).

#### 2.5.3. Immunogenicity Prediction

For candidate neoantigens passing expression and LC–MS/MS evidence filtering, immunogenicity was predicted using DeepImmuno (v1.2). The model takes the peptide sequence and corresponding HLA allele as input and outputs a score reflecting the likelihood of eliciting a T-cell response. Following the original DeepImmuno model and previous reports, a score > 0.7 was used to define highly immunogenic candidates [35]. Candidate peptides with lengths of 8 or 11 amino acids bypass the DeepImmuno evaluation and are assigned an ‘NA’ score, but are strictly retained in the final output for evaluation via other multi-omics evidence.

#### 2.5.4. Sequence Similarity to Experimentally Validated Neoantigens

Our group previously developed the dbPepNeo series of immunogenic peptide databases [36,37]. To assess sequence similarity between candidate neoantigens and experimentally validated immunogenic neoantigens, homology searches were performed using BLAST+ blastp (v2.16) [38] against dbPepNeo2.0 (accessed on 10 October 2025) [37]. Given the short length of candidate peptides, the blastp-short task was used with an E-value ≤ 200, and low-complexity filtering (SEG) and composition-based statistics were disabled to avoid excessive penalties. Because randomly matched E-values for 8–11-mer peptides are intrinsically high even for perfect alignments, this relatively permissive E-value threshold was used only as an initial heuristic filter to avoid excessive false negatives [39]. For each peptide, key statistics from the best hit (identity percentage, alignment length, E-value, bitscore) were recorded, and hits with ≥ 80% identity were used as evidence for prioritization. Crucially, because dbPepNeo2.0 consists exclusively of experimentally validated short immunogenic peptides, enforcing full-length sequence coverage would be overly restrictive in this short-peptide comparison context. Instead, a stringent sequence identity threshold (≥80%) combined with alignment length was used to capture potentially shared core immunologically relevant sequence features.

### 2.6. Visualization and Statistical Analysis

To evaluate transcriptome-level expression differences between tumor and matched normal samples and to support the biological interpretation of candidate neoantigens, gene-level differential expression analysis was performed using DESeq2 (v1.48.2) [40]. Gene-level estimated counts aggregated from kallisto transcript-level quantification with tximport (Section 2.5.1) were used as input. Differential testing was conducted under a paired tumor–normal design with the patient as a blocking factor. Multiple testing was corrected using the Benjamini–Hochberg procedure to obtain adjusted *p* values (padj) [41].

Volcano plots were used to visualize differential expression results, with padj < 0.05 as the significance threshold and |log2FoldChange| > 1 as the effect-size threshold. Genes passing expression filtering and peptide–HLA prescreening were linked to the candidate neoantigen list by gene symbols. Heatmaps were generated from the DESeq2 variance-stabilizing transformation (VST) expression matrix, followed by gene-wise (row-wise) z-score normalization, and visualized using pheatmap (v1.0.13). Hierarchical clustering was performed using Euclidean distance and complete linkage.

### 2.7. Implementation and Reproducibility of SVNeoPP

To improve reproducibility and portability, we packaged the above analysis steps into the SVNeoPP tool. SVNeoPP uses Snakemake for modular orchestration and automated execution. Snakemake explicitly represents dependencies among workflow steps as a directed acyclic graph (DAG) and automatically determines which tasks to run based on the provided inputs and available intermediate results. When a workflow is interrupted or inputs are updated, only the affected steps are recomputed, enabling resumable execution and incremental updates while minimizing unnecessary recomputation.

SVNeoPP comprises four functional modules, which are decomposed into interdependent rules that can be executed in parallel on local multicore machines or cluster/cloud environments. To ensure environment consistency and portability, dependency management is handled via Conda/Mamba (https://github.com/mamba-org/mamba, accessed on 16 March 2026), and containerized environments using Docker (https://www.docker.com/) and/or Singularity images are provided for key software components. Outputs are organized by project, sample, and module, with key intermediate files retained and summary tables generated for quality control, result tracking, and reruns. SVNeoPP is publicly available at https://github.com/Wanyang-AH/SVNeoPP (accessed on 16 March 2026).

### 2.8. Benchmarking and Comparative Analysis

To evaluate the performance of SVNeoPP for SV-derived neoantigen prioritization, we benchmarked it against NeoSV (v0.04) using a ranking-based top-*N* comparison framework, a commonly used and direct strategy in neoantigen prioritization studies [42]. Because NeoSV typically yielded a smaller number of candidate peptides, its candidate output size was used as the reference, so that the two methods could be compared under the same candidate budget. This strategy was intended to improve comparability and to assess the relative ability of the two methods to preferentially retain high-priority candidates.

At the peptide level, the analysis included candidate peptides predicted by both SVNeoPP and NeoSV. Within each method, candidates were prioritized according to NetChop_Score and Internal_Risk_norm. Three candidate cutoffs (top 50%, top 75%, and top 100%) were applied, and under these cutoffs the distributions of NetChop_Score and Internal_Risk_norm between the two methods were compared using two-sided Mann–Whitney U tests. This analysis was used to evaluate which method preferentially retained candidate peptides with higher predicted terminal cleavage potential and lower internal cleavage risk under matched candidate budgets.

At the peptide–HLA pair level, the analysis included candidate records that passed the NetChop pre-screening threshold and contained complete binding-affinity-related fields. For each candidate peptide, the optimal HLA record was retained based on NetMHCpan_Rank_EL and mhcflurry_presentation_score for EL-based and presentation-based analyses, respectively. The same three candidate cutoffs were applied, and under these cutoffs the distributions of EL rank and presentation score between the two methods were compared using two-sided Mann–Whitney U tests.

Overall, this framework enabled benchmarking of SVNeoPP and NeoSV at both the peptide level and the peptide–HLA pair level, providing a unified basis for comparing their prioritization performance in identifying high-priority SV-derived neoantigen candidates.

**Table 1:** Software and versions used in the SVNeoPP workflow.

Module	Software	Version	Description
Module 1	Fastp [18]	v1.0.1	Quality control and adapter/low-quality trimming of raw sequencing FASTQ reads.
	BWA-MEM [19]	v0.7.19	Aligns DNA sequencing reads to the human reference genome.
	STAR [43]	v2.7.11b	RNA-seq genome alignment for downstream BAM-based analyses.
	SAMtools [20]	v1.22.1	Format conversion, sorting and indexing of BAM files.
	GATK [21]	v4.6.2.0	Standard processing and refinement of BAM files.
	SvABA [22]	v1.2.0	Somatic structural variant calling.
	AnnotSV [23]	v3.4.6	Functional annotation of structural variants at the gene level.
	Custom Python scripts (SVNeoPP)	Python 3.11	Integration of SV annotations and generation of candidate mutant peptides.
Module 2	OptiType [24]	v1.3.5	HLA-I typing from sequencing data.
	Kallisto [30]	v0.51.1	Transcript quantification for TPM filtering and tximport-based gene-level counts for DESeq2.
Module 3	FragPipe [33]	v23.1	Database search and quantification of MS data to validate candidate neoantigen peptides.
Module 4	NetChop [26]	v3.1	Prediction of proteasomal cleavage probabilities along peptide sequences.
	NetMHCpan [28]	v4.1b	Prediction of peptide–HLA-I binding affinity.
	MHCflurry [29]	v2.1.5	Prediction of peptide–HLA-I binding affinity and presentation scores.
	DeepImmuno [35]	v1.2	Deep-learning prediction of immunogenicity for 9–10mer peptides.
	BLASTp [38]	v2.16	Local-align candidates to dbPepNeo2.0; use significant hits as clues.

**Table 2:** Reference resources and database builds used in SVNeoPP.

Category	Resource/Build	Version/Release	Description
Reference genome	GRCh38 (hg38, UCSC) [44]	accessed on 16 August 2025	Reference genome used for somatic variant calling and RNA-seq alignment.
Gene annotation	GENCODE gene annotation (GTF) [45]	gencode.v47.annotation.gtf	Transcript/CDS annotation used for SV mapping.
Annotation database	gene_annotation.db (gffutils) [46]	Built from gencode.v47.annotation.gtf on 17 September 2025	gffutils database built from the gene annotation GTF, used to map SVs to transcripts/CDSs and to support peptide generation.
Human proteome	UniProtKB (Homo sapiens, UP000005640) [47]	Reviewed proteome, accessed on 2 September 2025	Human reference proteome used as the MS search database.
Contaminants database	cRAP [48]	accessed on 2 September 2025	Common contaminant proteins used in MS searches.
Neoantigen peptides	Custom neoantigen FASTA	generated in this study	Custom FASTA of candidate neoantigen peptides generated in this study and included in the MS search database.
Experimentally validated database	dbPepNeo2.0 [37]	accessed on 10 October 2025	Assess the similarity of candidate neoantigen peptides against the database.

## 3. Results

In this study, we developed SVNeoPP, an end-to-end workflow for predicting and prioritizing SV-derived neoantigens using multi-omics data. SVNeoPP integrates SV-derived peptide generation, peptide–HLA binding prediction, and multi-dimensional features to support evidence-informed prioritization. Benchmarking against NeoSV showed overall improved performance in candidate generation scale, and integrated quality-related metrics. We further applied SVNeoPP to a real HCC cohort, characterizing the SV-derived neoantigen landscape and peptide–HLA binding patterns, evaluating multi-dimensional evidence support, and presenting the final prioritized candidates.

### 3.1. Overview of the SVNeoPP Workflow

**Figure 2:**
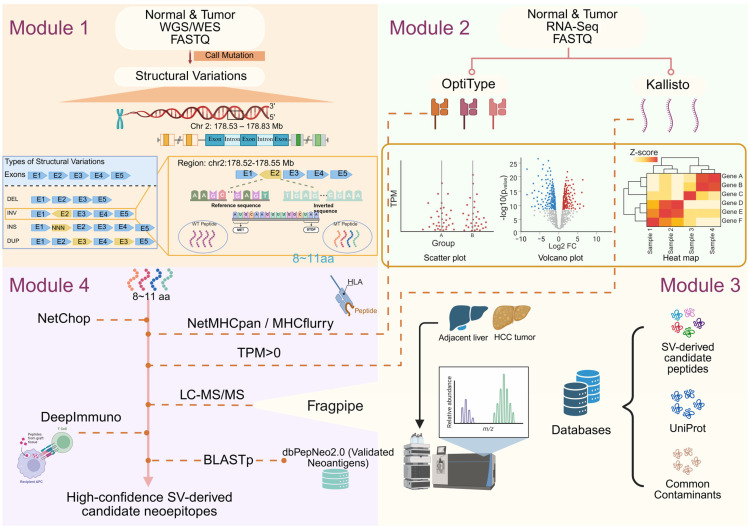
Overview of the SVNeoPP workflow for structural variation–derived neoantigen discovery. SVNeoPP integrates paired tumor–normal WGS, tumor RNA-seq, and LC–MS/MS proteomics data to identify and prioritize SV-derived neoantigens. Although the workflow is primarily designed for WGS, it can in principle be adapted for WES input. The workflow consists of four modules: (**1**) SV detection and SV-derived peptide reconstruction; (**2**) HLA class I typing and gene expression quantification; (**3**) proteomics-based evidence support for candidate peptides; and (**4**) multi-feature integration for filtering and prioritization. The output is a ranked set of high-confidence SV-derived neoantigen candidates.

The workflow of SVNeoPP is shown in Figure 2, consisting of four functional modules. Module 1: SV Calling and Peptide Generation. This module takes paired tumor–normal WGS data as input for quality control, alignment, and somatic SV calling. Altered CDSs and protein sequences are then reconstructed under transcript-structure constraints to generate SV-derived peptides. Although the workflow is primarily designed for WGS, it can also be applied to WES data; however, because WES has lower sensitivity for SV detection due to its limited capture space, results should be interpreted with caution [49]. Module 2: HLA Typing and Expression Quantification. RNA-seq data are used to infer individual HLA alleles and quantify transcripts at the gene level. They provide inputs for downstream binding-affinity prediction and expression-based filtering, and support differential expression analysis and visualization (e.g., volcano plots and clustered heatmaps). Module 3: Proteomics-based Evidence Support. A personalized search database is constructed by combining candidate peptides with a reference protein database, followed by LC–MS/MS database searching. This provides proteomics-level spectral evidence supporting candidate neoantigen identification. Module 4: Candidate Filtering and Prioritization. This module integrates features including peptide processing potential, peptide–HLA binding affinity, gene expression, MS evidence, immunogenicity scores, and sequence similarity to experimentally validated neoantigens to filter and prioritize SV-derived neoantigens. To facilitate use, SVNeoPP organizes these modules into a reproducible workflow and produces standardized outputs.

### 3.2. Comparative Evaluation of SV-Derived Neoantigen Prediction

To provide a clear and structured overview of the methodological differences between the two workflows, we summarized their key features, multi-omics integration capabilities, and prioritization strategies as shown in Table 3. We then benchmarked the two workflows from three complementary perspectives: candidate set scale, robustness of retained candidates in the NetChop dimension, and score distributions related to binding and presentation.

**Figure 3:**
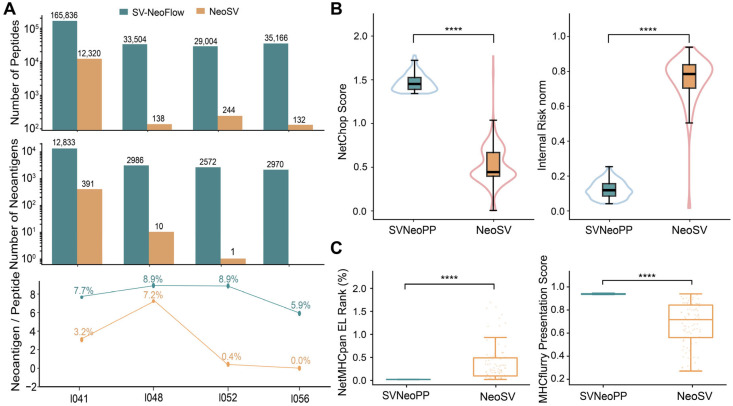
Comparison of SVNeoPP and NeoSV for SV-derived peptides and candidate neoantigen in HCC. (**A**) Comparison of numbers of SV-derived peptides, predicted candidate neoantigens, and the percentage of candidate neoantigens among peptides for each HCC sample. (**B**) Comparison of processing-related features at the top 100% cutoff under the ranking-based top-N framework: NetChop_Score and Internal_Risk_norm. (**C**) Comparison of binding-related metrics at the top 100% cutoff after retaining the optimal HLA record for each1 candidate peptide: NetMHCpan_Rank_EL and mhcflurry_presentation_score. Statistical significance was assessed using two-sided Mann–Whitney U tests; **** *p* ≤ 0.0001.

For the candidate set scale, as shown in Figure 3A–C, SVNeoPP generated 2.9 × 10^4^–1.7 × 10^5^ SV-derived peptides per sample, while NeoSV generated 1.3 × 10^2^–1.2 × 10^4^ peptides, indicating a substantial difference in scale between the two workflows. For predicted candidate neoantigens, SVNeoPP produced 2.6 × 10^3^–1.3 × 10^4^ candidates across samples (median ~3.0 × 10^3^), whereas NeoSV yielded fewer than 400 candidates in three samples and none in one. Using the “neoantigen count/peptide count” ratio as a proxy for conversion rate, SVNeoPP achieved 5.9–8.9% across the four samples, whereas NeoSV achieved 0–7.2%. Overall, in these proof-of-concept samples, SVNeoPP substantially expanded the search space without a notable decrease in conversion rate.

Given the substantial difference in output scale between the two workflows, we compared antigen-processing features under a ranking-based top-N framework, using the NeoSV candidate size as the reference. At the top 100% cutoff, SVNeoPP candidates exhibited a higher NetChop_Score distribution than NeoSV (median: 1.4528 vs. 0.4406; two-sided Mann–Whitney U test, *p* = 1.58 × 10^−97^), indicating improved terminal-processing suitability. SVNeoPP candidates also showed a lower Internal_Risk_norm distribution (median: 0.1024 vs. 0.7723; *p* = 2.43 × 10^−93^), suggesting a reduced risk of undesirable internal cleavage. Similar trends were observed under the top 75% and top 50% cutoffs (Supplementary Figures S1 and S2). These results indicate that, under matched candidate budgets, SVNeoPP preferentially retained peptides with improved terminal-processing suitability and lower internal cleavage risk.

For peptide–HLA binding metrics, the same framework was applied after retaining the optimal HLA record for each peptide. At the top 100% cutoff, SVNeoPP-derived peptides showed lower NetMHCpan_Rank_EL values than NeoSV (median: 0.005 vs. 0.218; *p* = 7.64 × 10^−29^) and higher mhcflurry_presentation_score values (median: 0.98344 vs. 0.73908; *p* = 1.55 × 10^−28^), indicating stronger predicted binding affinity and presentation potential. Consistent patterns were also observed under top 75% and top 50% cutoffs (Supplementary Figures S1 and S2).

In summary, in the proof-of-concept samples, SVNeoPP generated a richer set of SV-derived peptides and candidate neoantigens than NeoSV while demonstrating improved antigen-processing and HLA-binding properties, providing a more favorable starting point for downstream multi-omics evidence integration and immunogenicity evaluation.

### 3.3. Landscape and Characteristics of SV-Derived Candidate Neoantigens

**Figure 4:**
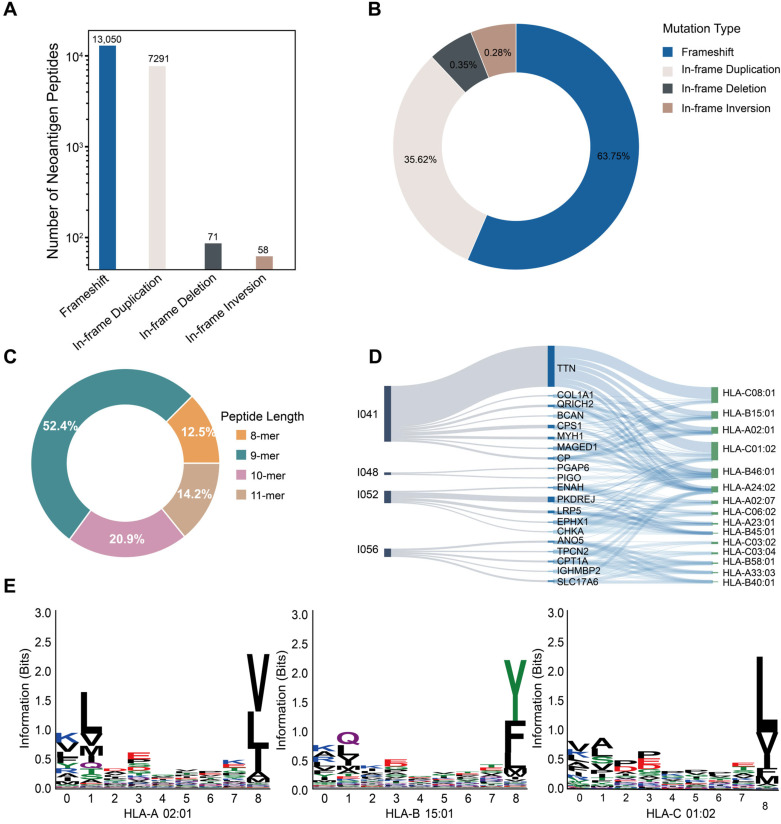
Landscape and characteristics of structural-variant-derived candidate neoantigen peptides in HCC samples identified by SVNeoPP. (**A**) Counts of candidate peptides categorized by mutation type. (**B**) Proportional composition of mutation types based on tool-native definitions. (**C**) Distribution of peptide lengths. (**D**) Sankey diagram illustrating the distribution of peptide–HLA pairs across sample, source gene, and HLA allele layers; the gene layer displays the top 20 genes ranked by pooled occurrence. Link width is proportional to the number of peptide–HLA pairs. (**E**) Sequence logos for 9-mer peptides generated using Logomaker (v0.8.7).

For SV-derived candidate neoantigens identified by SVNeoPP in HCC samples, we summarized the source types of peptides based on the annotation and classification systems for SV coding consequences (Figure 4A,B). The candidate peptides were primarily derived from frameshift-related categories and finer subtypes of in-frame events, with duplication-related in-frame (DUP_in-frame) events accounting for a relatively high proportion, while subtypes such as DEL_in-frame and INV_in-frame events contributed only a small fraction (<1%). In terms of peptide length, candidates were predominantly 9-mer peptides, which is consistent with classical HLA-I preferences, while the overall length distribution was broader, spanning 8–11 amino acids (Figure 4C). For comparison, a similar analysis of NeoSV-derived candidates is presented in Supplementary Figure S3.

We further examined the distribution of SVNeoPP-predicted peptide–HLA pairs across samples, source genes, and HLA alleles. As shown in Figure 4D, *TTN* was among the top 20 most frequent source genes. To externally validate this association, we searched IEDB for *TTN*-derived HLA ligands identified in HCC from publicly available immunopeptidomics studies, along with donors’ HLA typing information (Supplementary Table S4). *TTN-derived ligands* were observed in *multiple donors*, and their *HLA alleles *(e.g., *HLA-A*02:01, *HLA-A24:02*, and *HLA-C*06:02) overlapped with those in Figure 4D [50], supporting the SVNeoPP predictions.

We next assessed allele-specific binding preferences at the sequence level. We selected the representative alleles with the highest number of associated source genes from the HLA loci (*HLA-A02:01*, *HLA-B15:01*, and *HLA-C01:02*) and extracted their corresponding 8–11-mer peptide sequences for motif analysis. As 9-mer peptides were the most abundant length, Figure 4E shows sequence logos for 9-mer peptides, while logos for other lengths are provided in Supplementary Figure S4A–C. The results revealed distinct amino-acid enrichment patterns at key positions across alleles: HLA-A02:01 preferred hydrophobic residues at N-terminal anchor positions and showed enrichment of V/L at the C-terminal position; HLA-B15:01 displayed pronounced enrichment of Y/F at the C-terminal position; and HLA-C01:02 was enriched for L at the C-terminal position, with additional residue preferences at several N-terminal positions. Together, these findings *suggest* that peptide–HLA pairings output by SVNeoPP exhibit allele-associated binding preferences at the sequence level.

### 3.4. Multi-Omics Support for Prioritizing SV-Derived Candidate Neoantigens

**Figure 5:**
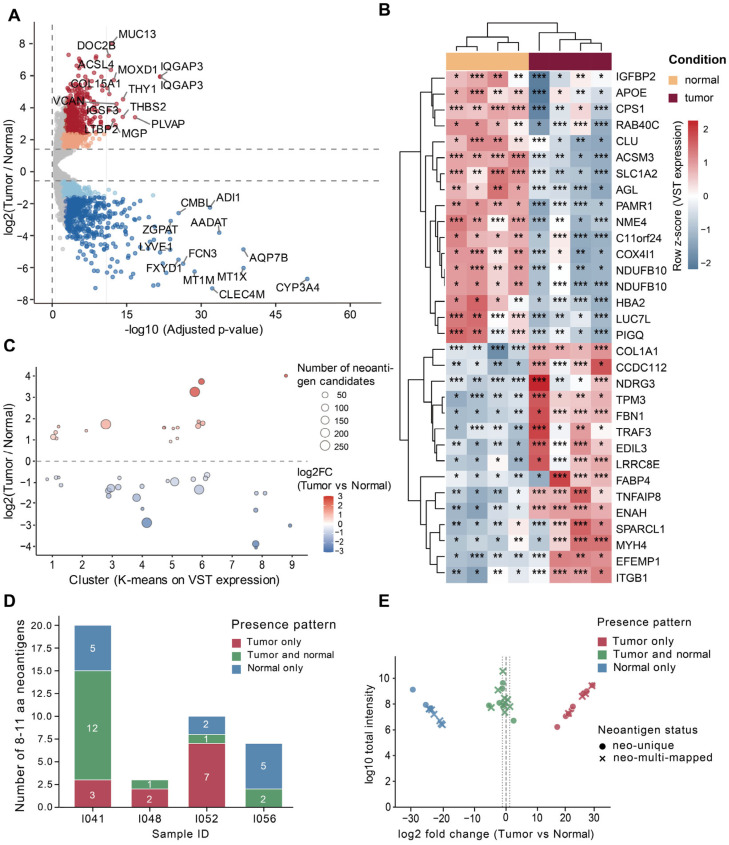
Multi-omics integration to filter and support SV-derived candidate neoantigens. (**A**) Differential expression analysis of candidate genes in tumor and matched normal samples. (**B**) Heatmap of hierarchical clustering of 32 differentially expressed genes across samples. (**C**) Bubble plot of the distribution of candidate-related genes across multiple expression-pattern clusters. (**D**) Categorization of candidate neoantigens based on MS detection patterns in tumor and normal samples. (**E**) Quantification and comparison of intensity changes between tumor and matched tissues for the three peptide groups; point shape indicates mapping type (unique vs. multi-mapped). * *p* ≤ 0.05, ** *p* ≤ 0.01, *** *p* ≤ 0.001.

To further filter and prioritize SV-derived neoantigens, we incorporated transcriptomic and proteomic evidence. Gene-level differential expression analysis was performed using RNA-seq data from tumors and matched normal samples. As shown in Figure 5A, among the 15,412 tested genes, numerous genes were significantly up- or downregulated, reflecting widespread transcriptome remodeling in tumors. By integrating these results with the SV-neoantigen candidate gene list, genes with RNA-seq expression evidence were systematically screened. Using thresholds of padj < 0.05 and |log2FoldChange| > 1, 32 differentially expressed genes were identified (Supplementary Table S5), including 17 previously reported HCC-associated genes (e.g., *CPS1* and *COL1A1*) [51,52,53]. Additionally, hierarchical clustering of the expression levels of these 32 genes across samples revealed two distinct high-expression modules corresponding to tumor and normal samples (Figure 5B), indicating that this gene set can distinguish tumor from matched normal samples.

To further examine genome-wide expression patterns in relation to differential-expression direction and candidate burden, we performed an exploratory K-means clustering on the VST-transformed transcriptome-wide expression matrix as a background partitioning for visualization. We selected K = 9 based on comparisons with neighboring values (K = 7, 9, and 11), which showed qualitatively consistent enrichment and mapping patterns (Supplementary Figure S5). Standard model-selection metrics (Elbow, Silhouette, and Gap statistics) yielded inconsistent recommendations (Supplementary Figure S6) and were therefore not used as the primary criteria for K selection. Because the strictly defined differentially expressed gene set was too sparse to reveal broader expression modules, we used a relaxed cutoff (padj < 0.1 and |log2FoldChange| > 0.5) only for this exploratory visualization, yielding 47 genes (Supplementary Table S6). As shown in Figure 5C, these genes were distributed across multiple clusters, with both upregulated and downregulated genes represented. Candidate burden was unevenly distributed, with a small subset of genes linked to more candidate pairings. This analysis provides an exploratory view of how candidate-associated genes are distributed across background expression-pattern clusters, rather than serving as a primary basis for statistical inference or downstream prioritization.

Furthermore, we incorporated LC–MS/MS proteomics database search results to provide proteomics-level evidence for candidate neoantigens. Peptides with MS support were categorized based on their detection patterns in paired samples: Tumor-only, Tumor-and-normal, and Normal-only (Figure 5D). Based on tumor specificity, we preferentially retained Tumor-only peptides and removed Normal-only peptides, while Tumor-and-normal peptides were considered secondary candidates. Moreover, we quantified and contrasted the intensity changes between tumor and matched samples for the three groups (Figure 5E). Tumor-only and Normal-only peptides exhibited opposite trends in relative change direction, suggesting MS detectability reflects sample type. In contrast, Tumor-and-normal peptides clustered near zero-change, indicating similar detectability in both conditions and highlighting the need for additional assessment of tumor specificity using immunogenicity and sequence similarity.

### 3.5. Immunogenicity and Sequence Similarity for Prioritizing SV-Derived Neoantigens

**Figure 6:**
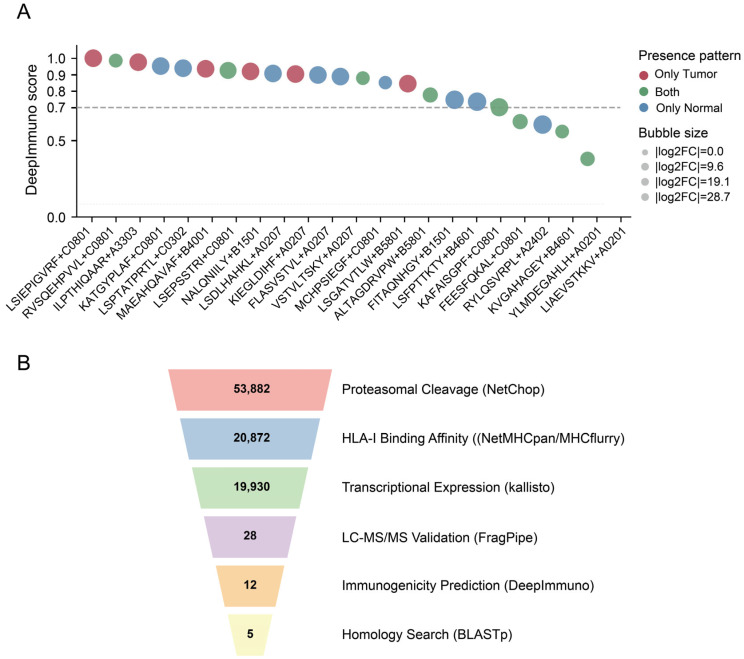
Immunogenicity prediction and tiered filtering strategy for candidate neoantigens. (**A**) DeepImmuno scoring of MS-supported candidate peptide–HLA pairs. The dashed line indicates the DeepImmuno score threshold of 0.7 used for prioritization. Colors indicate detection pattern, and bubble size encodes |log2FC(Tumor vs. Normal)| based on proteomics intensity changes. (**B**) Funnel plot of the stepwise filtering process for SV-derived candidate peptides, with numbers indicating non-redundant peptide sequences aggregated across samples.

Building on the candidate neoantigens supported by tumor-side transcript expression and proteomic evidence, we further evaluated their potential immunogenicity at the peptide–HLA pair level (Figure 6A). Since the same peptide–HLA pair may recur across different cases, for entries with multiple records, the highest immunogenicity score was used as the representative value. Using a DeepImmuno > 0.7 threshold for prioritization and excluding “Normal only” entries, 12 high-scoring candidates were identified. Figure 6A shows the magnitude of DeepImmuno intensity differences between tumor and normal for these high-scoring candidates, helping to interpret their proteomic trends. Notably, five candidate neoantigens (8 mer or 11 mer) fell outside the length constraints of the DeepImmuno model and were assigned ‘NA’ for immunogenicity, though they remained supported by other layers of evidence. We further filtered the candidate peptides using the experimentally validated neoantigens dataset from the dbPepNeo2.0 database, with a filtering threshold of similarity ≥ 80%. This resulted in five high-priority candidates: “NALQNIILY”, “FEESFQKAL”, “LSEPSSTRI”, “MCHPSIEGF”, and “KIEGLDIHF”. Subsequently, we validated these peptides using the Gene Expression Profiling Interactive Analysis 2 (GEPIA2) [54] and Human Protein Atlas (HPA) [55] databases. Among them, the peptide “NALQNIILY” corresponded to *POSTN*, which exhibited exceptionally high tumor-specific expression in HCC in our datasets. This tumor-specific expression not only reduces the potential toxicity risk to normal tissues but also increases the immunological visibility of the mutant peptide, making it a promising neoantigen candidate.

To visually illustrate the stepwise narrowing from large-scale predictions to high-priority entries, we sequentially filtered the unique candidate neoantigen sequences (merged and deduplicated across the four samples) along key evidence dimensions, counting the number of candidates retained at each step (Figure 6B). Using the SV-derived peptides, SVNeoPP first performed prescreening based on proteasomal processing and HLA binding, then further reduced the candidate set by incorporating transcript expression evidence. LC–MS/MS detection evidence served as the primary bottleneck for further narrowing the candidates: after removing peptides detected only in normal samples, the number of candidates decreased significantly. Finally, within the MS-supported subset, we prioritized candidates by integrating DeepImmuno immunogenicity scores and homology searches against dbPepNeo2.0, resulting in five high-priority candidates.

## 4. Discussion

Predicting SV-derived neoantigens can provide additional targets, especially in tumors with low mutational burden. However, existing approaches remain limited in the traceability of sequence derivation, multi-evidence integration, and end-to-end reproducibility [7,56]. To address these challenges, we developed SVNeoPP, a workflow for predicting SV-derived neoantigens.

SVNeoPP introduces three main innovations as follows: (1) Traceable sequence reconstruction. Under a multi-transcript context, SVNeoPP reconstructs altered transcripts and coding sequences, converting SV events into translatable sequences for candidate peptide generation. This approach helps mitigate the impact of breakpoint uncertainty and annotation errors on downstream inference. (2) Multi-evidence, end-to-end filtering and prioritization. We integrate multi-omics evidence (WGS, RNA-seq, and LC–MS/MS proteomics) to evaluate and prioritize candidates in a tiered manner, linking variant calling and sequence derivation with immunologic evaluation in an evidence-driven selection loop. (3) End-to-end reproducibility. SVNeoPP is orchestrated with Snakemake, enabling execution from raw inputs with traceable parameters, ensuring reproducible outputs and facilitating stable operation and extension in multi-omics contexts.

Using HCC multi-omics data, we systematically demonstrated an evidence-driven, end-to-end workflow in SVNeoPP. This design allows candidates to progressively converge from “computationally supported” predictions to a high-priority set with stronger evidence support and more interpretable prioritization criteria. Benchmarking against the existing SV neoantigen workflow, NeoSV, shows that SVNeoPP generates an order of magnitude more SV-derived candidate peptides, substan-tially expanding the candidate pool. Importantly, further comparison of key immunological features reveals that the advantage of SVNeoPP is not solely due to “producing more”; its candidates show potential for stronger performance in antigen processing and peptide–HLA binding, reflecting the enrichment of peptides that are predicted to have higher presentation potential by widely used models.

Beyond the current implementation, SVNeoPP can be further extended by integration with linear pangenome references. Previous studies have demonstrated that linear pangenomes outperform single linear reference genomes in structural variant detection [57]. Under this framework, more comprehensive SV discovery may further expand the candidate space of SV-derived neoantigens and associated clinical biomarkers.

Despite the advancements offered by SVNeoPP, several limitations warrant further improvement. First, sequence reconstruction remains influenced by upstream uncertainty. Although we provide a traceable reconstruction strategy under a multi-transcript context, breakpoint errors, transcript-selection biases, and potential post-transcriptional regulation may affect the authenticity and completeness of candidate peptides. Incorporating more refined transcript-structure evidence—such as full-length transcript characterization via long-read sequencing—could improve the accuracy of SV-associated coding sequence reconstruction. Second, LC–MS/MS proteomics evidence primarily serves as supportive evidence and as a filtering signal, and cannot replace immunopeptidomics-based validation of antigen presentation. Direct evidence from immunopeptidomics is still needed to confirm whether candidate peptides are truly presented as HLA-bound ligands, which is the direction we are currently pursuing. Third, this study primarily considered peptides presented by HLA class I, whereas SVs may also generate longer peptides which are suitable for HLA class II presentation. Future work could incorporate an HLA-II module. Finally, the demonstration and benchmarking here were conducted on a small HCC cohort as a proof of concept, focusing on workflow runnability and evidence-integration strategy rather than generalizing SV-derived neoantigen burden or distribution. Future studies should systematically evaluate the robustness of this workflow in larger cohorts and across additional cancer types in order to further strengthen the reliability and generalizability of the conclusions.

## 5. Conclusions

In summary, SVNeoPP provides a reusable analytical workflow with a transparent, interpretable, tiered prioritization strategy for SV-derived neoantigens. As a complement to SNV/small-indel-based neoantigen workflows, it enables SV-focused candidate derivation and multi-evidence integration that accounts for breakpoint complexity, thereby expanding the pool of potential neoantigen peptides. In the future, supported by large cohorts and functional validation experiments, SVNeoPP could further strengthen the evidence chain for SV-derived neoantigens, offering more reliable candidates for neoantigen vaccine development and personalized immunotherapy.

## Figures and Tables

**Table 3:** Comparison of key methodological features and prioritization strategies between SVNeoPP and NeoSV.

Feature/Dimension	SVNeoPP	NeoSV
Workflow Engine (Reproducibility)	Snakemake (End-to-end automated)	Python package
Starting Input Data	Raw FASTQ (WGS/WES, RNA-seq) and LC-MS/MS	Pre-called SV VCF/BEDPE
Supported SV Types	DEL, DUP, INS, INV, BND (Fusion)	DEL, DUP, TRA, h2hINV, t2tINV
Transcript Reconstruction Strategy	Isoform-aware, database-backed (gffutils)	Heuristic (Selects the transcript with the longest CDS)
Antigen Processing Filter	NetChop (Terminal potential + Internal risk penalty)	No
MHC Binding Prediction	NetMHCpan and MHCflurry	NetMHCpan
RNA-seq Integration (Expression)	Yes (kallisto TPM and DESeq2 differential analysis)	No
Proteomic Validation (LC-MS/MS)	Yes (Personalized FragPipe database search)	No
Immunogenicity Prediction	Yes (DeepImmuno)	No
Homology Search (Validated targets)	Yes (BLASTp against dbPepNeo2.0)	No
Prioritization Strategy	Multi-dimensional evidence (Binding + Expression + MS + Immunogenicity + Homology)	Binding

## Data Availability

Tumor and matched normal WGS data from four hepatocellular carcinoma patients were obtained from our previously collaborated and published work from the Chinese Human Proteome Project (CNHPP) [17]. Transcriptome data are available in the Gene Expression Omnibus (GEO) under accession GSE124535. Mass spectrometry data are available via iProX under accession IPX0000937000 (http://www.iprox.org, accessed on 28 September 2022). The analysis code and SVNeoPP workflow is available at https://github.com/Wanyang-AH/SVNeoPP (accessed on 16 March 2026).

## Supplementary Materials

The following supporting information can be downloaded at https://www.mdpi.com/article/10.3390/biology15060492/s1: **Figure S1**: Comparison of SVNeoPP and NeoSV under the top 75% cutoff of the ranking-based top-N framework. Figure S2: Comparison of SVNeoPP and NeoSV under the top 50% cutoff of the ranking-based top-N framework. Figure S3: Landscape and characteristics of structural-variant-derived candidate neoantigen peptides predicted by NeoSV. Figure S4: Length-stratified sequence motifs for representative HLA class I alleles. Figure S5: Robustness of the exploratory visualization across different K-means cluster granularities. Figure S6: Evaluation of optimal cluster number (K) for K-means clustering. Table S1: Pearson correlation matrix of terminal and internal cleavage features. Table S2: Grid-based comparison of Internal_Risk parameter settings (α and β). Table S3: Sensitivity analysis of candidate retention under different NetChop prescreening thresholds. Table S4: *TTN*-derived HLA class I ligands reported in HBV-related HCC immunopeptidomics datasets. Table S5: List of 32 differentially expressed genes associated with SV-derived neoantigen candidates. Table S6: List of 47 SV-associated neoantigen candidate genes.
