# Supplementary material for: SVNeoPP: A Workflow for Structural-Variant-Derived Neoantigen Prediction and Prioritization Using Multi-Omics Data
Source: Biology (Basel). 2026 Mar 19;15(6):492. doi: 10.3390/biology15060492 (PMC13024079; doi:10.3390/biology15060492)
Supplement: Supplementary file 1 [file biology-15-00492-s001.zip › biology-4174254-supplementary.pdf]

Supplementary Information

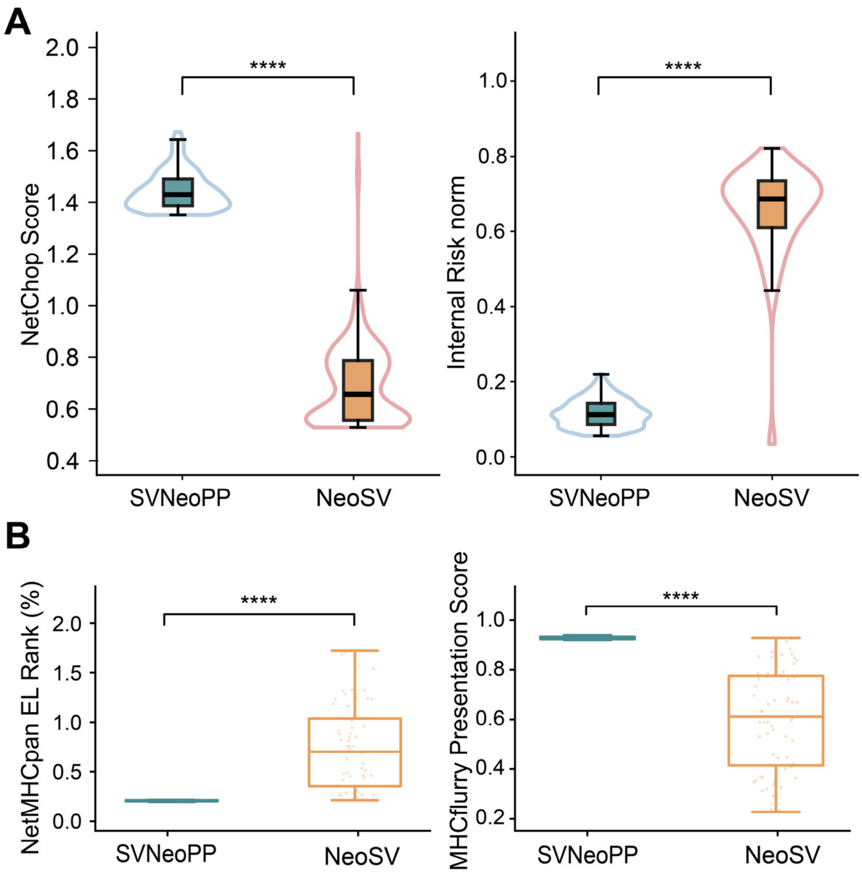

Supplementary Figure S1. Comparison of SVNeoPP and NeoSV under the top 75% cutoff of the ranking-based top-N framework.

(A) Comparison of processing-related features, including NetChop_Score and Internal_Risk_norm, between SVNeoPP and NeoSV. (B) Comparison of binding-related metrics, including NetMHCpan_Rank_EL and mhcflurry_presentation_score, after retaining the optimal HLA record for each candidate peptide.

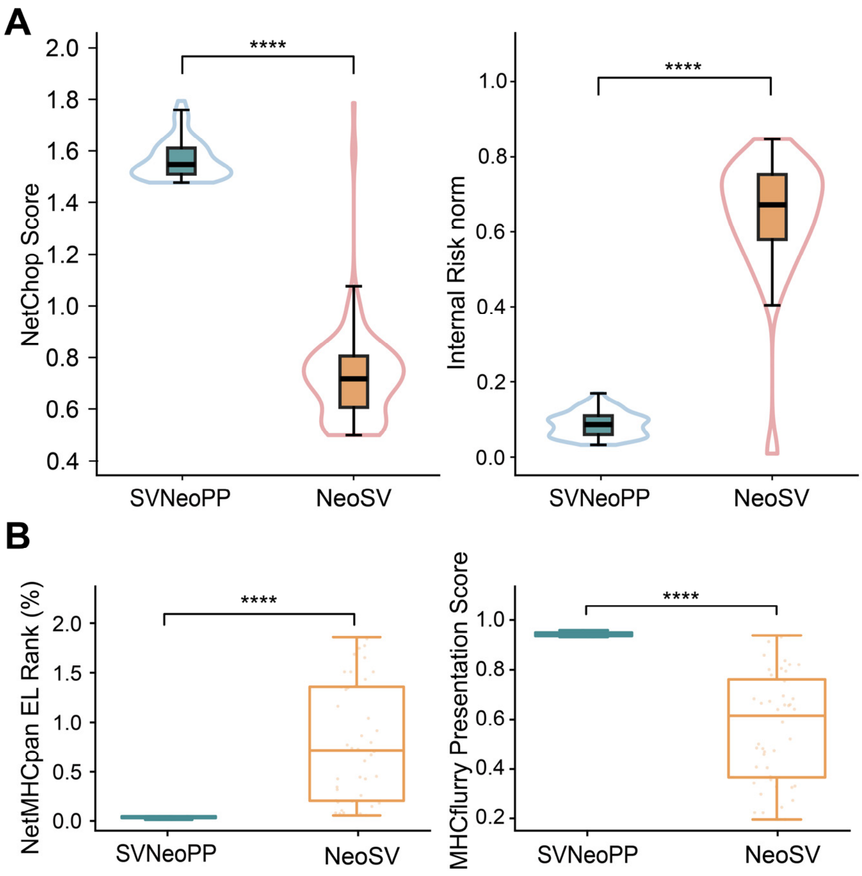

Supplementary Figure S2. Comparison of SVNeoPP and NeoSV under the top 50% cutoff of the ranking-based top-N framework.

(A) Comparison of processing-related features, including NetChop_Score and Internal_Risk_norm, between SVNeoPP and NeoSV. (B) Comparison of binding-related metrics, including NetMHCpan_Rank_EL and mhcflurry_presentation_score, after retaining the optimal HLA record for each candidate peptide.

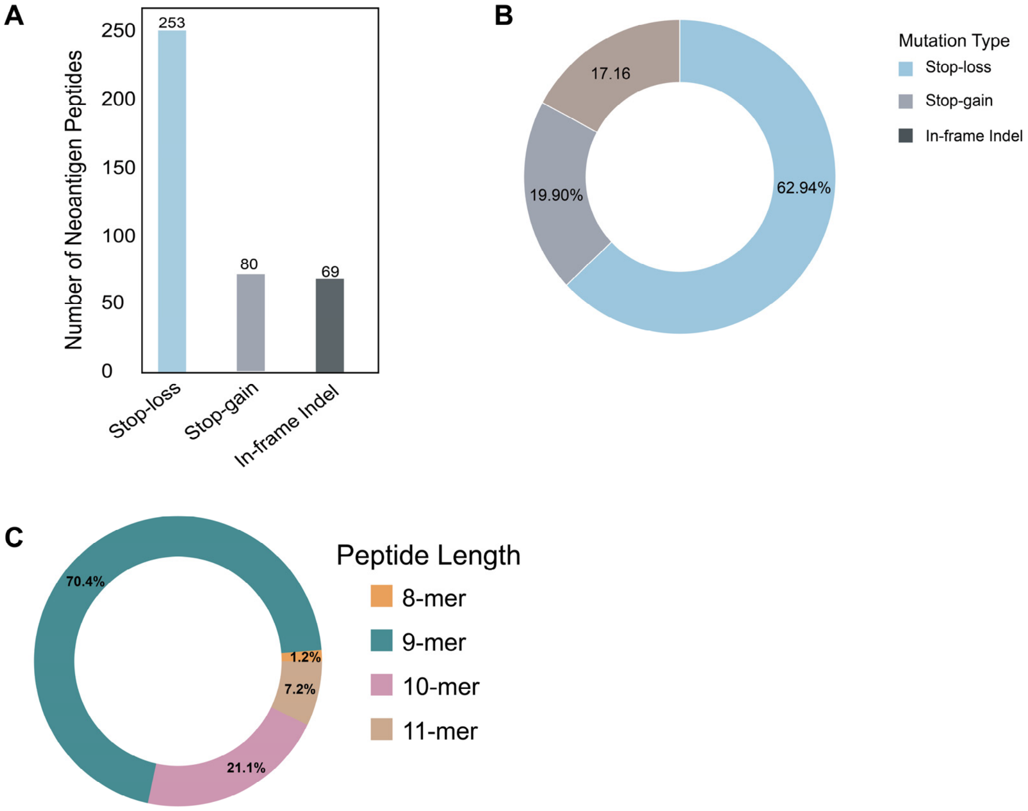

Supplementary Figure S3. Landscape and characteristics of structural-variant-derived candidate neoantigen peptides predicted by NeoSV.

(A) Counts of candidate neoantigen peptides predicted by NeoSV across different mutation types. (B) Proportional composition of mutation types, including Stop-loss, Stop-gain, and In-frame Indel. (C) Distribution of candidate peptide lengths (8–11 mer), with 9-mers representing the largest proportion.

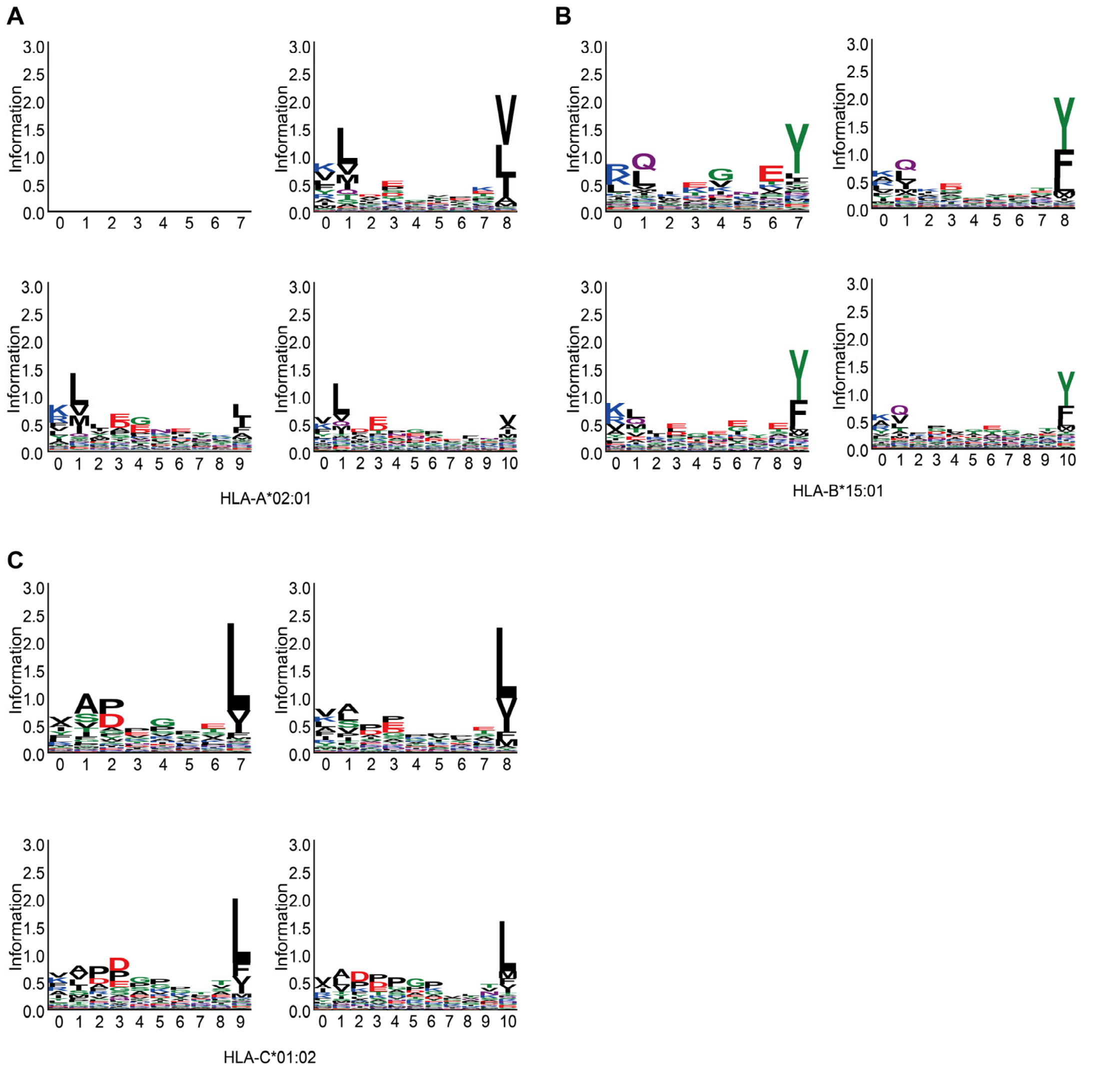

Supplementary Figure S4. Length-stratified sequence motifs for representative HLA class I alleles. Sequence logos depict the binding specificities of (A) HLA-A*02:01, (B) HLA-B*15:01, and (C) HLA-C*01:02, stratified by peptide lengths of 8, 9, 10, and 11 amino acids. Note that the motif for the HLA-A*02:01 8-mer group is not shown due to the limited sample size (N = 3). Logos display information content (bits) and were generated using Logomaker.

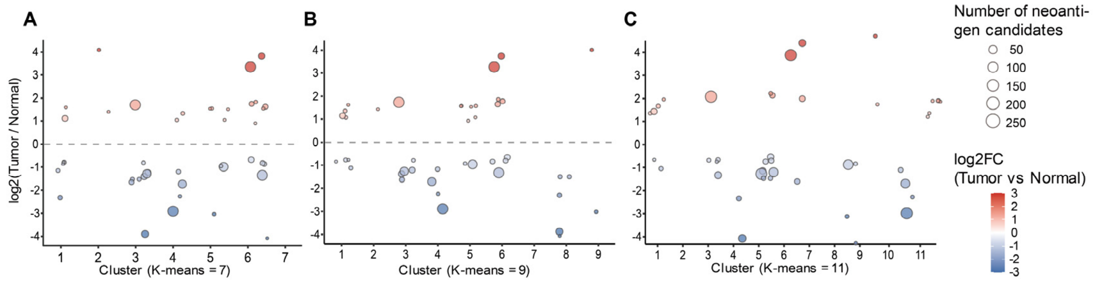

Supplementary Figure S5. Robustness of the exploratory visualization across different K-means cluster granularities.

Bubble plots mapping the 47 neoantigen-related genes to the background expression clusters when partitioned into (A) K=7, (B) K=9, and (C) K=11 clusters. The y-axis represents the differential expression direction (log2FoldChange), and the bubble size indicates the number of neoantigen candidates associated with each gene. The consistent enrichment pattern of neoantigen candidates—primarily within specific downregulated clusters—across varying background granularities demonstrates that the observed biological trends are robust and not artificially driven by the specific heuristic choice of K=9.

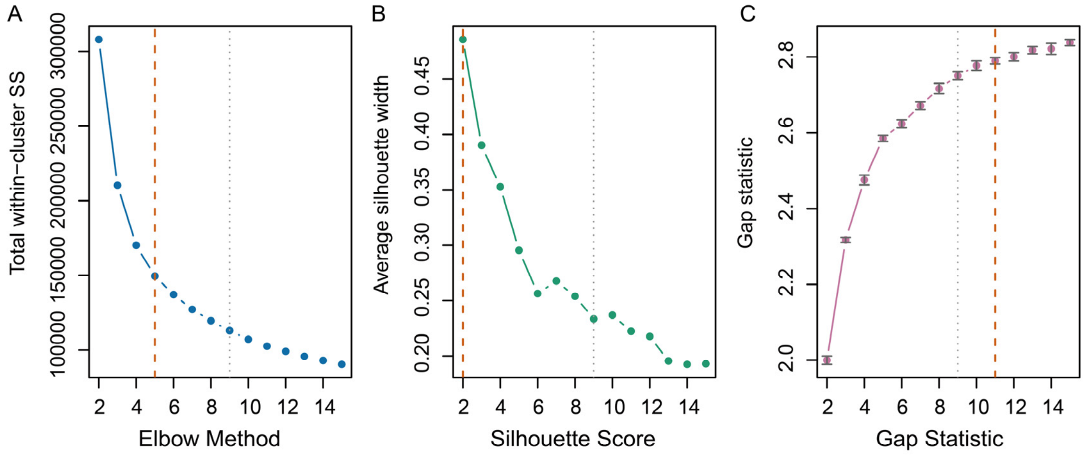

Supplementary Figure S6. Evaluation of optimal cluster number (K) for K-means clustering. (A) The Elbow method, (B) Silhouette score, and (C) Gap statistic calculated across a range of K values (K=2 to 15). The orange dashed lines indicate the diverse mathematical optimums suggested by each individual metric (K=5, K=2, and K=11, respectively). The gray dotted lines indicate K=9, which was heuristically selected for the exploratory visualization. The highly conflicting recommendations across metrics highlight the necessity of employing an empirical choice to balance visual resolution and pattern discovery in high-dimensional transcriptomic data.

Supplementary Table S1. Pearson correlation matrix of terminal and internal cleavage features.

Features	N_score	C_score	max_internal_score	internal_cleavage_count
N_score	1.000	-0.022	-0.061	-0.113
C_score	-0.022	1.000	-0.078	-0.118
max_internal_score	-0.061	-0.078	1.000	0.525

internal_cleavage_count	-0.113	-0.118	0.525	1.000

Note: Correlations were calculated from 263,510 SV-derived candidate peptides. Weak correlations between terminal scores and internal features support modeling internal cleavage risk as an independent penalty, whereas the moderate correlation between max_internal_score and internal_cleavage_count supports down-weighting the count term to avoid over-penalization.

Supplementary Table S2. Grid-based comparison of Internal_Risk parameter settings (α and β).

α (alpha)	β (beta)	Mean Risk	SD	25th Percentile	Median (50th)	75th Percentile
0.5	0.1	0.549	0.104	0.522	0.581	0.616
0.5	0.3	0.788	0.176	0.713	0.814	0.899
0.5	0.5	1.026	0.253	0.934	1.037	1.178
1.0	0.1	0.980	0.176	0.939	1.038	1.087
**1.0**	**0.3**	**1.218**	**0.242**	**1.144**	**1.283**	**1.375**
1.0	0.5	1.456	0.315	1.321	1.515	1.658
1.5	0.1	1.410	0.248	1.353	1.498	1.566
1.5	0.3	1.648	0.312	1.567	1.742	1.848
1.5	0.5	1.886	0.381	1.762	1.984	2.134

Note: The selected parameter combination (α = 1.0, β = 0.3) provided an intermediate and empirically balanced Internal_Risk distribution among the tested settings and was therefore used in this study.

Supplementary Table S3. Sensitivity analysis of candidate retention under different NetChop prescreening thresholds.

NetChop_Score threshold	Retained peptides (n)	Retained peptides (%)
≥ 0.3	146,568	55.6
≥ 0.5	53,882	20.4
≥ 0.7	17,573	6.7

Note: Sensitivity analysis was performed using alternative NetChop prescreening thresholds (0.3, 0.5, and 0.7). Percentages were calculated relative to the total number of SV-derived peptides evaluated (n = 263,814).

Supplementary Table S4. *TTN*-derived HLA class I ligands reported in HBV-related HCC immunopeptidomics datasets.

IEDB Epitope ID	Donor	Stage	Peptide(AA)	Sample / APC	Method & IP	MHC Restr.	MHC Alleles
954603	15	Unknown	KEPPIFRKK(9)	Liver hepatocyte; Direct ex vivo	/ cellular MHC/MS; W6/32	HLA-I	HLA-A*23:01; HLA-A*30:01; HLA-B*35:01; HLA-B*53:01; HLA-C*04:01
	17	Cancer Stage II	KEPPIFRKK(9)	Liver hepatocyte; Direct ex vivo	/ cellular MHC/MS; W6/32	HLA-I	HLA-A*01:01; HLA-A*02:01; HLA-B*08:01; HLA-B*14:01; HLA-C*07:01; HLA-C*08:02
2383484	17	Cancer Stage II	KTVELDVAD(9)	Liver hepatocyte; Direct ex vivo	/ cellular MHC/MS; W6/32	HLA-I	HLA-A*01:01; HLA-A*02:01; HLA-B*08:01; HLA-B*14:01; HLA-C*07:01; HLA-C*08:02
2382374	10	Cancer Stage II	KALKSDIGQY(10)	Liver hepatocyte; Direct ex vivo	/ cellular MHC/MS; W6/32	HLA-I	HLA-A*02:01; HLA-A*32:01; HLA-B*37:01; HLA-B*44:02; HLA-C*05:01; HLA-C*06:02
2389539	16	Cancer Stage III	STATFEAHISGF(11)	Liver hepatocyte; Direct ex vivo	/ cellular MHC/MS; W6/32	HLA-I	HLA-A*24:02; HLA-B*18:01; HLA-B*38:02; HLA-C*07:01; HLA-C*07:02

(data sourced from de Beijer MT et al., JHEP Reports, 2022).

Supplementary Table S5. List of 32 differentially expressed genes associated with SV-derived neoantigen candidates.

Gene ID	BaseMean	log_2FoldChange	IfcSE	Stat	P-value	P-adj	Gene Symbol	Label
ENSG00000005187	1727.704	-3.7614	0.47793	-7.87018	3.54E-15	8.81E-13	ACSM3	ACSM3
ENSG00000007392	807.1556	-1.3021	0.361727	-3.59969	0.000319	0.003434	LUC7L	LUC7L
ENSG00000007541	670.3173	-1.40989	0.423264	-3.33099	0.000865	0.007369	PIGQ	PIGQ
ENSG00000021826	37118.48	-2.8429	1.031013	-2.75739	0.005826	0.03008	CPS1	CPS1
ENSG00000101079	437.8255	1.464571	0.564239	2.595655	0.009441	0.042535	NDRG3	NDRG3
ENSG00000103202	1510.676	-1.45693	0.392209	-3.71468	0.000203	0.002459	NME4	NME4
ENSG00000108821	1482.295	1.421015	0.338865	4.193458	2.75E-05	0.000505	COL1A1	COL1A1
ENSG00000110436	1193.896	-3.93206	0.593891	-6.62084	3.57E-11	3.89E-09	SLC1A2	SLC1A2
ENSG00000115380	717.7768	4.244984	0.712311	5.959451	2.53E-09	1.79E-07	EFEMP1	EFEMP1
ENSG00000115457	3015.042	-2.29769	0.832205	-2.76097	0.005763	0.029808	IGFBP2	IGFBP2
ENSG00000120885	75686.8	-1.26768	0.362928	-3.49291	0.000478	0.004681	CLU	CLU
ENSG00000130203	164938.2	-1.76101	0.680418	-2.58812	0.00965	0.043289	APOE	APOE
ENSG00000131143	5663.375	-1.22024	0.367213	-3.32297	0.000891	0.007527	COX4I1	COX4I1
ENSG00000131323	689.9401	1.346707	0.468323	2.875596	0.004033	0.023258	TRAF3	TRAF3
ENSG00000140990	711.1564	-1.56733	0.371068	-4.22383	2.40E-05	0.000454	NDUFB10	NDUFB10
ENSG00000143549	5376.983	1.318853	0.328106	4.0196	5.83E-05	0.000927	TPM3	TPM3
ENSG00000145779	364.2698	1.274288	0.344076	3.703506	0.000213	0.002529	TNFAIP8	TNFAIP8
ENSG00000149090	172.491	-2.96387	0.46536	-6.369	1.90E-10	1.72E-08	PAMR1	PAMR1
ENSG00000150093	7562.545	1.070369	0.377048	2.838809	0.004528	0.025272	ITGB1	ITGB1
ENSG00000152583	636.6401	1.539622	0.591501	2.602907	0.009244	0.041986	SPARCL1	SPARCL1
ENSG00000154380	1155.451	2.934307	0.47016	6.241078	4.35E-10	3.62E-08	ENAH	ENAH
ENSG00000162688	1049.232	-1.68245	0.346183	-4.86	1.17E-06	3.76E-05	AGL	AGL
ENSG00000164176	43.84959	4.998917	1.016379	4.918359	8.73E-07	2.92E-05	EDIL3	EDIL3

ENSG00000164221	78.15141	1.137678	0.401255	2.835297	0.004578	0.025509	CCDC112	CCDC112
ENSG00000166147	1046.497	3.377622	0.591097	5.714155	1.10E-08	6.54E-07	FBN1	FBN1
ENSG00000170323	206.693	3.628675	1.085809	3.34191	0.000832	0.007136	FABP4	FABP4
ENSG00000171017	155.7841	1.266498	0.436044	2.904518	0.003678	0.021767	LRRC8E	LRRC8E
ENSG00000171067	936.4478	-1.35001	0.383367	-3.52147	0.000429	0.004306	C11orf24	C11orf24
ENSG00000188536	1840.607	-5.51448	0.883051	-6.2448	4.24E-10	3.55E-08	HBA2	HBA2
ENSG00000197562	312.2692	-1.0379	0.388503	-2.67153	0.007551	0.036304	RAB40C	RAB40C
ENSG00000264424	82.5078	4.05923	1.267749	3.20192	0.001365	0.010365	MYH4	MYH4
ENSG00000291930	711.1564	-1.56733	0.371068	-4.22383	2.40E-05	0.000454	NDUFB10	NDUFB10

***Note*:** Genes were filtered based on strict differential expression criteria (padj < 0.05, |log_2FoldChange| > 1) between tumor and normal samples.

Supplementary Table S6. List of 47 SV-associated neoantigen candidate genes.

Gene ID	Gene Symbol	Cluster	$\log_2$FoldChange	P-adj	N_neo
ENSG00000005187	ACSM3	8	-3.7614	8.81E-13	75
ENSG00000110436	SLC1A2	8	-3.93206	3.89E-09	1
ENSG00000149090	PAMR1	9	-2.96387	1.72E-08	3
ENSG00000188536	HBA2	8	-5.51448	3.55E-08	54
ENSG00000154380	ENAH	6	2.934307	3.62E-08	265
ENSG00000115380	EFEMP1	5	4.244984	1.79E-07	9
ENSG00000166147	FBN1	6	3.377622	6.54E-07	54
ENSG00000164176	EDIL3	2	4.998917	2.92E-05	2
ENSG00000162688	AGL	3	-1.68245	3.76E-05	21
ENSG00000140990	NDUFB10	8	-1.56733	0.000454	7
ENSG00000291930	NDUFB10	8	-1.56733	0.000454	7
ENSG00000108821	COL1A1	3	1.421015	0.000505	274
ENSG00000143549	TPM3	1	1.318853	0.000927	1
ENSG00000141027	NCOR1	1	-0.96073	0.001982	1
ENSG00000103202	NME4	3	-1.45693	0.002459	50
ENSG00000205155	PSENEN	6	-0.92082	0.002483	18
ENSG00000145779	TNFAIP8	5	1.274288	0.002529	2
ENSG00000007392	LUC7L	3	-1.3021	0.003434	41
ENSG00000171067	C11orf24	3	-1.35001	0.004306	130
ENSG00000120885	CLU	4	-1.26768	0.004681	13
ENSG00000170323	FABP4	9	3.628675	0.007136	2
ENSG00000007541	PIGQ	6	-1.40989	0.007369	226
ENSG00000131143	COX4I1	1	-1.22024	0.007527	6
ENSG00000264424	MYH4	7	4.05923	0.010365	164
ENSG00000171017	LRRC8E	5	1.266498	0.021767	1
ENSG00000131323	TRAF3	6	1.346707	0.023258	27
ENSG00000150093	ITGB1	1	1.070369	0.025272	5
ENSG00000164221	CCDC112	2	1.137678	0.025509	1
ENSG00000168916	ZNF608	5	0.806324	0.026829	1
ENSG00000141552	ANAPC11	3	-0.90592	0.028105	4
ENSG00000110321	EIF4G2	1	0.803494	0.029089	2
ENSG00000103148	NPRL3	6	-0.95707	0.029614	18
ENSG00000115457	IGFBP2	8	-2.29769	0.029808	12
ENSG00000021826	CPS1	4	-2.8429	0.03008	622
ENSG00000197562	RAB40C	5	-1.0379	0.036304	3
ENSG00000143742	SRP9	1	0.869516	0.038187	38
ENSG00000090266	NDUFB2	1	-0.88991	0.039998	7
ENSG00000163870	TPRA1	6	-0.78102	0.041123	40
ENSG00000152583	SPARCL1	6	1.539622	0.041986	3
ENSG00000101079	NDRG3	6	1.464571	0.042535	19
ENSG00000130203	APOE	4	-1.76101	0.043289	125
ENSG00000105223	PLD3	1	-0.88898	0.043735	1

ENSG00000164180	TMEM161B	5	0.672192	0.056804	1
ENSG00000090565	RAB11FIP3	5	-1.07221	0.069457	164
ENSG00000152495	CAMK4	5	1.238465	0.072942	1
ENSG00000156804	FBXO32	5	1.274819	0.080542	2
ENSG00000163631	ALB	4	-2.24579	0.083349	2

Note: Candidate genes were identified using K-means clustering (K=9) and selected with relaxed thresholds (padj < 0.1, |log_2FoldChange| > 0.5) to capture broader expression patterns.
